# Sagittal spinal alignment in adolescent idiopathic scoliosis: a narrative review of pre- and post-treatment characteristics

**DOI:** 10.1530/EOR-2025-0263

**Published:** 2026-06-01

**Authors:** Yu Song, Yimin Yan, Cao Yang, Kun Wang

**Affiliations:** Department of Orthopedics, Union Hospital, Tongji Medical College, Huazhong University of Science and Technology, Wuhan, China

**Keywords:** adolescent idiopathic scoliosis, sagittal spinal alignment, brace treatment, posterior spinal fusion

## Abstract

- Adolescent idiopathic scoliosis (AIS) involves significant sagittal plane abnormalities, most consistently characterized by reduced thoracic kyphosis (TK), with compensatory changes in lumbar lordosis (LL) and cervical lordosis (CL).
- Brace treatment effectively controls coronal curve progression but commonly reduces TK and LL, potentially leading to a flatback deformity, while global sagittal balance is often maintained through spinal and pelvic compensation.
- Surgical intervention, particularly posterior spinal fusion, reliably improves sagittal alignment, with the most pronounced restoration of TK and CL observed in patients with preexisting sagittal malalignment.
- Despite regional alterations, global sagittal balance typically remains stable posttreatment, underscoring the body’s inherent compensatory mechanisms to maintain an energy-efficient upright posture.
- Pelvic parameters exhibit considerable individual variability but adapt to help sustain overall spinal equilibrium, with a tendency toward retroversion when compensating for spinal imbalances.
- Clinical management of AIS should integrate personalized, three-dimensional assessment and correction strategies, balancing coronal correction with the imperative of achieving optimal sagittal alignment to ensure favorable long-term outcomes.

## Introduction

Adolescent idiopathic scoliosis (AIS) is a complex spinal deformity characterized by lateral curvature, vertebral rotation, and sagittal spinal curvature disruption, with a Cobb angle >10° in the frontal plane (1, 2). AIS affects 2–3% of the general population, primarily between ages 10 and 16 (1, 3). Progressive untreated AIS may lead to increased back pain, respiratory symptoms, significant cosmetic concerns, and reduced quality of life (QoL) (2, 4, 5, 6). Therefore, early detection and appropriate management are essential to minimize long-term sequelae.

The significance of sagittal plane alignment in the diagnosis and treatment of AIS is well established, as alignment imbalance is a critical indicator for progression and prognosis (4, 7) and is directly associated with various complications and compromised QoL (8, 9, 10). We, therefore, aimed to review evidence on the sagittal profile in patients with AIS, investigate changes following conservative and surgical interventions, and summarize the strengths and limitations of current approaches, thereby providing insights for achieving comprehensive three-dimensional (3D) correction.

### Radiographic assessment for spinopelvic sagittal evaluation

**Figure 1:**
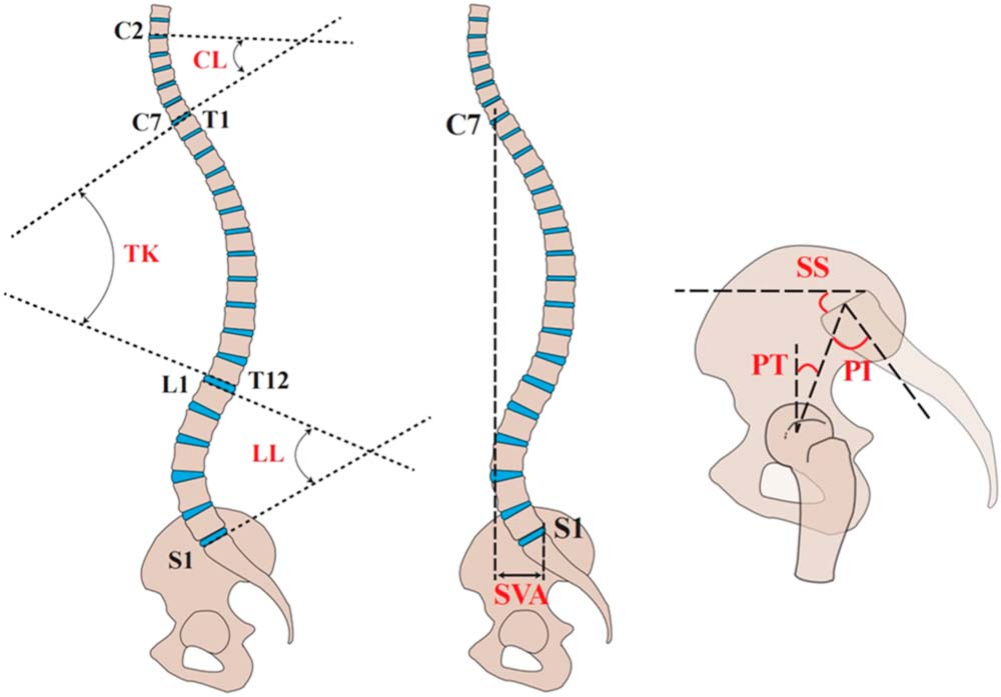
Spinopelvic parameters. CL, cervical lordosis; TK, thoracic kyphosis; LL, lumbar lordosis; SVA, sagittal vertical axis; SS, sacral slope; PT, pelvic tilt; PI, pelvic incidence.

Several radiographic parameters aid in measuring spinopelvic sagittal alignment (Table 1, Fig. 1) (11). Key among these are thoracic kyphosis (TK), lumbar lordosis (LL), and cervical lordosis (CL), which reflect the sagittal profile of their respective spinal segments, while the sagittal vertical axis (SVA) serves to assess overall spinal alignment. The spine is intricately linked with the pelvis. Pelvic morphology, characterized by parameters such as pelvic incidence (PI), pelvic tilt (PT), and sacral slope (SS), also plays a critical role in maintaining sagittal balance. Among them, PT and SS are dynamic parameters that vary with pelvic rotation and serve as important indicators of pelvic compensation under pathological conditions. In contrast, PI is an anatomical parameter that remains constant regardless of pelvic position; nevertheless, it determines the spatial orientation of the spine in relation to the pelvis (11) and governs the relationship between PT and SS through the equation PI = PT + SS (12, 13). The PI–LL mismatch is also a critical indicator of sagittal imbalance and correlates with clinical outcomes. Achieving a matching standard of PI–LL < 10° remains an important goal in spinal deformity correction (11).

**Table 1:** Common sagittal spinal-pelvic measurements: definitions and normal values.

Parameter	Definition	Normal values
Thoracic kyphosis	The angle between the superior endplate of T1 (occasionally T4 or T5) and the inferior endplate of T12	20°–40°
Lumbar lordosis	The angle between the superior endplate of L1 and the superior endplate of S1	40°–60°
Cervical lordosis	The angle between the inferior endplate of C2 and the inferior endplate of C7	20°–40°
Sagittal vertical axis (SVA)	The horizontal offset distance between the centroid of the C7 vertebral body and the posterosuperior corner of the S1 vertebral body, measured on standing full-spine lateral radiographs. By convention, SVA is positive (+) when the C7 plumb line falls anterior to the S1 corner and negative (−) when it falls posterior to it	<±50 mm
Pelvic incidence	The angle between a line perpendicular to the sacral plate at its midpoint and a line connecting this point to the bicoxofemoral axis	30–80°
Pelvic tilt	The angle between a vertical reference line (gravity line) and a line connecting the midpoint of the sacral plate to the bicoxofemoral axis	10°–15°
Sacral slope	The angle between the superior endplate of S1 and a horizontal reference line	30°–50°

Notably, pediatric spinopelvic sagittal characteristics differ from those of adults and exhibit a cascade effect strictly modulated by skeletal maturity stages (typically assessed using the Risser sign and triradiate cartilage status (TRC) or the more precise Sanders stage). Specifically, PI increases significantly during the initial growth peak (Risser 0, TRC open), which dictates a subsequent compensatory increase in proximal lumbar lordosis (PLL) during the second half of the pubertal growth spurt (Risser 0–1, TRC closed). Finally, distal thoracic kyphosis (DTK) increases toward skeletal maturity, thereby consolidating the adult sagittal profile. This sequential evolution underscores the pelvis’s role as the cornerstone of sagittal equilibrium and highlights the intricate reciprocal relationships among spinal regions (14, 15). Given that the peak of pelvic modification coincides with the high-risk period for AIS progression (16), assessing spinopelvic growth potential by integrating these skeletal maturity indicators is essential for accurately predicting the dynamic compensatory adjustments that evolve during growth in patients with AIS.

In clinical practice, the sagittal alignment of each spinopelvic unit is characterized by numerous parameters that capture morphological details. This review, however, focuses on the most representative sagittal measurements to summarize pre- and posttreatment sagittal profiles in patients with AIS, thereby providing a generalized understanding of key alignment characteristics reported in the literature.

### Characteristics of spinopelvic sagittal parameters in untreated AIS patients 

Patients with AIS exhibit sagittal imbalance early in the course of the disease (7) (Table 2). The thoracic spine is the primary site of AIS deformities, with most cases presenting with thoracic curves (17, 18, 19). Therefore, TK is a key parameter in sagittal plane research. Thoracic lordosis in AIS patients with thoracic curves has been extensively reported (7, 20, 21, 22, 23, 24, 25, 26, 27, 28, 29, 30, 31, 32, 33, 34, 35). This phenomenon may be attributed to vertebral rotation because AIS is a three-dimensional spinal deformity (36), which can be further exacerbated by relative anterior spinal overgrowth (RASO). RASO refers to the relatively faster growth of anterior vertebral bodies relative to posterior elements, which enhances axial rotational instability and, thus, promotes progressive deformity and loss of TK (37, 38). RASO represents a three-dimensional anatomical imbalance defined by an anterior–posterior length discrepancy. While influenced by a generalized vertebral osseous growth bias (37), this overgrowth is primarily driven by passive expansion of the anterior intervertebral discs (38). Rotational instability drives anterior axial unloading, which triggers passive disc expansion. As a result, the anterior column lengthens relative to the posterior column, transforming global thoracic kyphosis into a rotated apical lordosis (39). This mechanism establishes the biomechanical rationale for surgical strategies focused on three-dimensional correction via de-rotation and restoration of posterior column length. Notably, thoracic lordosis in AIS has been increasingly recognized as a regional phenomenon in recent years (7, 40, 41), and the overall morphology of the thoracic region exhibits both local hypokyphotic and hyperkyphotic zones (42, 43). Only considering the entire thoracic segment as a whole makes it difficult to comprehend its sagittal characteristics.

**Table 2:** Overview of spinopelvic sagittal morphology in untreated adolescent idiopathic scoliosis patients.

Spinal region	Characteristics	Clinical implications
Thoracic kyphosis	Patients with thoracic curves often exhibit a reduction in TK, which tends to be less pronounced in those with thoracolumbar/lumbar curves	Coronal plane deformities are coupled to the sagittal plane through vertebral rotation
Lumbar lordosis	Although the characteristics of LL exhibit considerable variation, it may demonstrate an increasing trend, particularly pronounced in patients with thoracolumbar/lumbar curves	Scoliosis occurring in the lumbar region may also be associated with marked vertebral rotation
		The sagittal alignment of the lumbar spine is significantly influenced by the thoracic spine and the pelvis
Cervical lordosis	CL may demonstrate a significant decrease or even progress to kyphotic reversal	Alterations in cervical lordosis represent a compensatory response to global spinal imbalance
Global alignment	The SVA typically remains within the normal range	Patients can maintain global sagittal balance through spontaneous spinal and pelvic compensation
Pelvis	Pelvic parameters demonstrate considerable individual variability, though elevated PI is frequently observed	The pelvis may exhibit a greater capacity for retroversion to compensate for imbalance, thereby maintaining upright posture through an energy-conserving mechanism

Sagittal alignment of the lumbar spine varies across studies. Significantly increased LL has been found in many cases (44, 45, 46, 47, 48) and is associated with complicating lumbar spondylolysis (49). However, some studies have also reported no significant change (20, 21, 23, 24, 50) or a reduction (51) in LL. On the one hand, LL exhibits a wider range and greater variability than TK (11). On the other hand, the morphology of LL is significantly influenced by other segments. The correlation between the lumbar and the thoracic region is well established, and patients with insufficient TK exhibit a pronounced reduction in LL (29, 52, 53). Sagittal changes in the thoracolumbar region can be transmitted through the junctional zone. The position and morphology of the pelvis can also significantly influence the configuration of LL via zygapophyseal joints, ligaments, and muscles (21, 36, 45). Therefore, changes in LL may depend on the overall spinopelvic sagittal profile and individual compensatory capabilities.

Notably, sagittal alignment in the thoracic and lumbar spine varies significantly across curve patterns. For example, patients presenting with thoracolumbar/lumbar curves commonly exhibit normal or even relatively higher thoracic kyphosis (7, 21, 23, 24, 47), along with more substantial lumbar lordosis (23, 36, 47). This may be explained by the more pronounced vertebral rotation in the lumbar region in these patients. However, this kind of coupling relationship remains independent of the coronal curve magnitude, despite different curve types (36). Nevertheless, sagittal alignment can be significantly altered by interventions aimed at correcting the coronal curve (54). Hence, careful monitoring of sagittal alignment remains essential and should not be overlooked.

The cervical spine is not primarily affected in AIS, but flattening of CL or change to cervical kyphosis in patients with AIS has been widely reported (32, 33, 34, 35, 47, 48, 52, 55, 56, 57, 58, 59, 60, 61, 62, 63, 64). Given the direct anatomical continuity between the cervical and thoracic spines, it is plausible that cervical alignment is influenced by the thoracic spine. Indeed, recent studies have confirmed that reduced TK can induce or exacerbate cervical kyphosis (32, 34, 52, 53, 55, 58, 61, 65, 66). However, no significant difference was observed in the incidence of cervical kyphosis between patients with thoracolumbar/lumbar curves and those with thoracic curves (59, 63). Moreover, cervical sagittal alignment is closely related to global sagittal parameters. Specifically, a shift in the SVA induces a backward-leaning posture, triggering compensatory forward positioning of the head and cervical spine that reduces CL or progresses to kyphosis (60, 65). Therefore, changes in cervical sagittal alignment in AIS should be considered part of overall trunk sagittal compensation (59, 62, 65). Further evaluation of overall sagittal balance by observing sagittal changes in the cervical spine at an early stage is warranted.

Despite regional sagittal deformity, the SVA in patients with AIS typically remains within normal limits (21, 50, 57, 61, 65, 66, 67). Several studies have utilized modified parameters of the SVA to assess patients with AIS and also found no significant abnormalities (32, 62, 68). It indicates that AIS patients can maintain global sagittal balance through spontaneous spinal and pelvic compensation (21, 52, 58). In addition, this reflects the body’s ability to dynamically maintain balance and an energy-efficient posture by keeping the gravity line within a conical area centered at the feet, a concept termed the ‘cone of economy’. When patients deviate from this cone, the ‘chain of balance’ relies on compensatory mechanisms in other body segments and increased muscle activity to offset sagittal imbalance. This elevated energy expenditure imposes a physiological burden and reduces QoL (69).

When focusing on the pelvis, current research lacks consensus. This discrepancy could be attributed to the considerable individual variability of pelvic parameters, which are influenced by a multitude of factors, including age and race (45, 70). Moreover, specific pelvic changes may be influenced by the sagittal alignment of the patient’s spine. However, a number of studies have found that PI in patients with AIS is significantly higher than that in normal adolescents (12, 21, 23, 47, 71, 72), which may suggest that the pelvis possesses a greater potential for retroversion as a compensatory mechanism to maintain upright postural balance while reducing muscle energy expenditure (45, 73).

### Impact of brace treatment on spinopelvic sagittal alignment

The conservative management of AIS includes brace treatment and physiotherapeutic scoliosis-specific exercises, among which brace treatment is the most commonly utilized and the only evidence-based effective nonoperative treatment (74, 75). Bracing is indicated for progressive AIS (+5° Cobb angle in 4–6 months) or AIS at high risk of progression (associated with Cobb angle and age at diagnosis) (16). The original design of the brace corrects spinal deformities by applying pressure and supportive forces, aiming to limit aggravation of AIS and minimize the risk of curve progression to final skeletal maturity. However, the design may also exert compressive effects on spinal sagittal alignment, leading to the flatback deformity. This effect may subsequently trigger chronic pain, pulmonary dysfunction, and accelerate intervertebral disc degeneration, thereby posing a threat to long-term QoL (9, 54). Although diverse brace types are available, thoracolumbosacral orthoses (TLSOs), such as the Cheneau brace and the Boston brace, represent the most commonly used designs in current clinical practice. The following section will focus on TLSOs to discuss the effects of bracing on sagittal spinal alignment (Table 3).

**Table 3:** Overview of the impact of brace treatment on spinopelvic sagittal alignment.

Spinal region	Characteristics	Clinical implications
Thoracic kyphosis	TK usually significantly decreases	The brace applies posterior pressure to the rib hump, thereby translating the trunk anteriorly
Lumbar lordosis	LL may decrease and is correlated with TK	Brace-induced shear forces along the spinal column influence the sagittal alignment of the lumbar spine
		Alterations in LL may serve as a compensatory mechanism for the reduced TK
Cervical lordosis	CL remained unchanged or decreased	Brace-induced shear forces along the spinal column not only influence cervical alignment but may also affect the development of the cervical articular facets
Global alignment	SVA generally remains stable, but may progress anteriorly in some cases	The posterior-directed force applied by the brace on the rib hump can induce anterior translation of the spine, while the spinal column retains a degree of self-adjusting capacity
Pelvis	Pelvic morphology is generally stable, albeit with substantial interindividual variation	Alterations in PI are associated with the specific bracing force application strategy
		To maintain overall global balance, the pelvis may undergo a degree of posterior tilt, manifesting as an increase in PT

**Figure 2:**
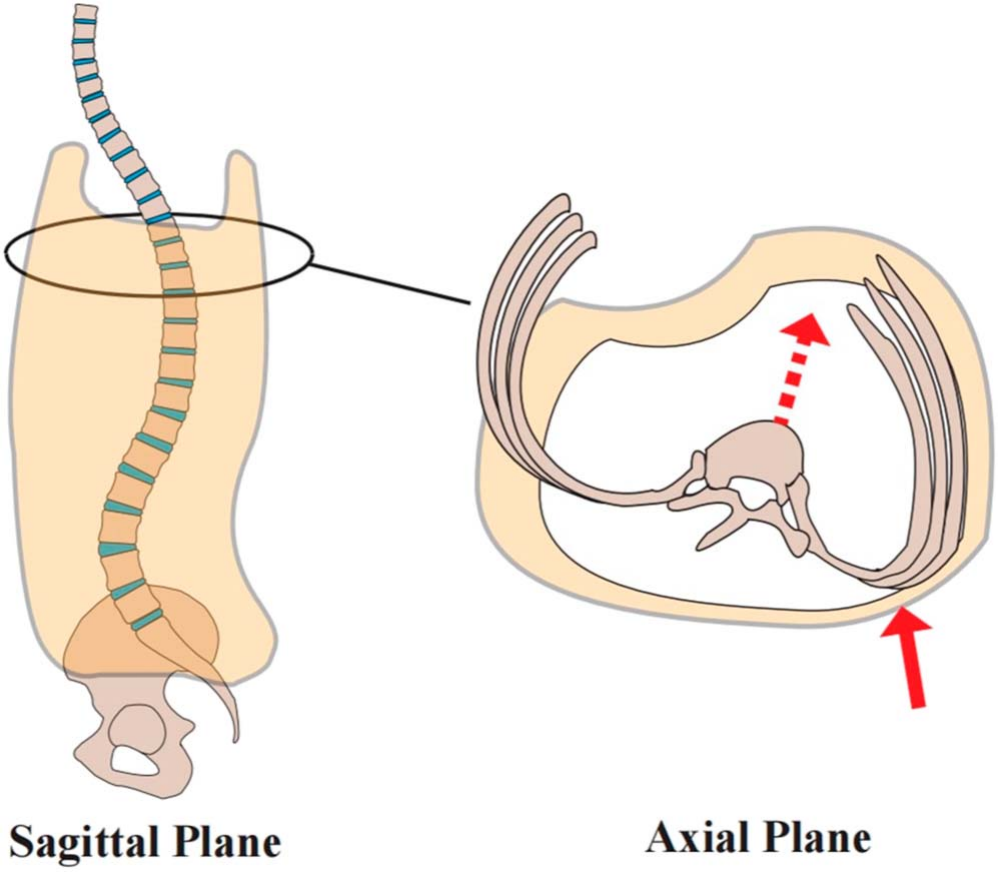
The posterior pressure exerted by the brace to the rib hump induces an anterior translation of the thoracic trunk.

Studies have identified loss of TK with bracing (9, 51, 54, 76, 77, 78, 79, 80, 81, 82), while patients with preexisting hypokyphosis typically do not exhibit significant changes therein (77, 80). This effect results from posterior pressure exerted by the brace on the rib hump (Fig. 2), which translates the trunk anteriorly (9, 78, 80, 81). This explains why the change in curvature is concentrated in the lower thoracic region (77, 80, 83), as this area serves as the usual point of force application for the brace.

Bracing may also lead to a significant reduction in LL (9, 51, 54, 76, 77, 78, 80, 81, 82, 83). On the one hand, this may be attributable to shear forces generated by the brace along the spinal column (9); additionally, braces equipped with ventral support significantly reduce LL (84). On the other hand, a significant correlation between LL and TK has been observed during brace treatment (54, 80), with reductions in LL also compensating for insufficiencies in TK.

CL showed a reduction (9, 76) or no change (77) following brace treatment. Although the cervical spine is not directly affected by the brace, given its intrinsically high mobility and the absence of a stabilizing mechanism, it may also be involved in the process of shear forces produced by the brace along the spinal column (76, 85). This can lead to CL flattening during treatment. Forces applied by the brace may also influence the development of the cervical articular facets, resulting in continuous reduction of CL (9, 76).

Due to the self-adjusting capacity of the spine, global spinal alignment may remain constant during brace treatment. Consequently, patients exhibit no significant change in SVA (9, 77, 79, 80). However, if the posterior-directed force applied by the brace on the rib hump deformity is pronounced, it may result in an anterior shift of SVA (54, 81), suggesting overall anterior inclination of the spine – a phenomenon that may persist beyond orthotic discontinuation (54).

Commonly, pelvic morphology tends to remain unchanged during brace treatment (9, 77, 80). However, the exact situation is also highly variable. Alterations in PI have been observed in some studies and are correlated with the magnitude of pressure applied by the brace to the pelvis: PI decreases when the force is applied to the caudal part of the sacrum and front of the iliac crest and increases with pressure applied to the cranial part of the sacrum and front of the iliac crest (78, 81). Some studies also observed pelvic retroversion with an increased PT (51, 78, 81). This adaptation was correlated with the patient’s PT out of brace: patients with initially low PT exhibited a proportional increase, suggesting a compensatory mechanism to normalize overall sagittal alignment, including pelvic orientation and cephalic positioning (51).

Regarding other brace types, the traditional Milwaukee brace has been largely phased out of clinical practice due to its poor compliance. The Milwaukee brace may lead to a significant reduction in TK and LL (86, 87, 88), and the neck ring thereof may stimulate the mandible, causing a trunk ‘upward extension’ reflex and straightening the spine (87). Soft braces are a flexible alternative, aiming to provide postural support with minimal restriction of movement. Within this category, elastic orthotic belts significantly reduce TK (87), while some other braces, such as the SpineCor (89), Spinaposture (90), and the anisotropic textile brace (91), have demonstrated favorable efficacy in the maintenance of sagittal balance. Nighttime bracing is an effective alternative intervention for patients who refuse full-time bracing (92, 93), and a marginal increase in TK has been observed, suggesting a significantly lower risk of developing flatback deformity compared to full-time bracing (94, 95). Although some of these braces demonstrate advantages in sagittal stability, the potential insufficiency of corrective forces compared to common TLSOs braces in the coronal plane should also be considered.

### Impact of surgical intervention on spinopelvic sagittal alignment

**Figure 3:**
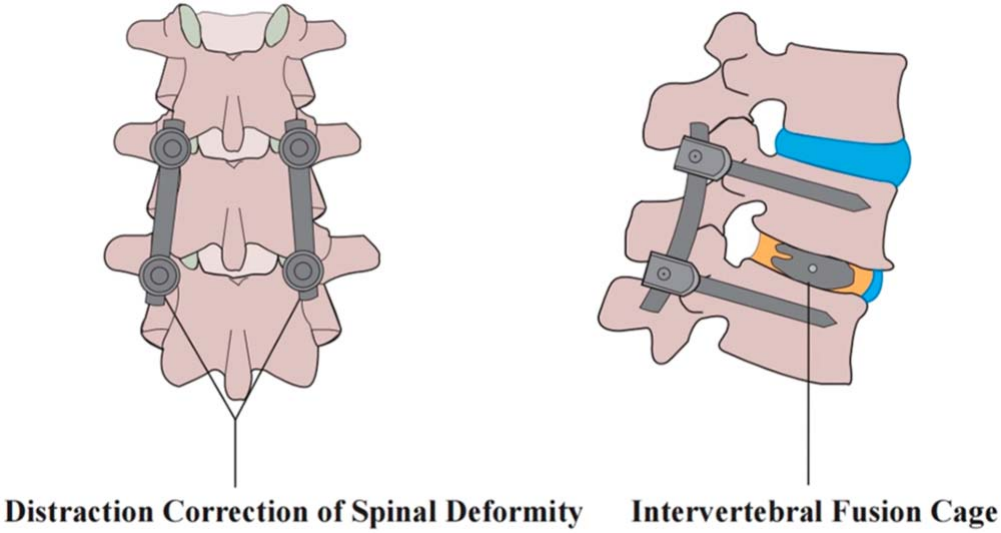
Posterior lumbar interbody fusion (PLIF) with pedicle screws.

Surgical intervention is indicated for severe AIS in skeletally immature patients with structural thoracic curves exceeding 40° or with evidence of curve progression (96, 97, 98). Spinal fusion is the most commonly utilized surgical treatment modality in clinical practice and can be classified into posterior fusion (PSF), anterior fusion (ASF), and combined fusion (96). PSF is the most widely used technique in clinical practice (99). The underlying principle involves posterior pedicle-screw instrumentation, which provides segmental fixation that, combined with pre-contoured rod translation or de-rotation and direct vertebral rotation, achieves three-dimensional correction of AIS and durable stabilization through PSF (Fig. 3). Recently, attention has turned toward the impact of surgical interventions on sagittal alignment, since patients with postoperative sagittal misalignment following AIS surgery tend to have an unfavorable prognosis (100, 101) (Table 4).

**Table 4:** Overview of the impact of surgical intervention on spinopelvic sagittal alignment.

Spinal region	Characteristics	Clinical implications
Thoracic kyphosis	Significant restoration of TK is observed, with more pronounced improvement in patients with preoperative sagittal malalignment	Surgical treatment typically leads to a significant improvement in TK
Lumbar lordosis	LL is typically maintained or increased postoperatively, and changes have been shown to be closely correlated with TK	Improvements in the thoracic region are, to some extent, biomechanically transmitted to the lumbar spine through the thoracolumbar junction and proximal lumbar segments
Cervical lordosis	Surgical intervention significantly improves cervical sagittal alignment, consistent with the restoration of TK	Cervical sagittal alignment demonstrates a compensatory improvement in response to the correction of other spinal regions
Global alignment	Postoperative SVA remains stable or shows improvement	Surgical interventions influence regional realignment, while compensatory mechanisms across spinal segments and the pelvis maintain the overall sagittal balance
Pelvis	Pelvic morphology and position generally remain stable, although retroversion may occasionally occur	Postoperative pelvic changes are generally minimal, although retroversion may occasionally occur to compensate for an increase in LL

PSF surgery has been associated with postoperative loss of or inadequate improvement in TK (10, 50, 99, 102, 103, 104, 105, 106, 107, 108, 109). However, a growing body of evidence in recent years indicates that surgical intervention can effectively restore TK and correct sagittal spinal deformity (32, 33, 34, 35, 48, 52, 56, 57, 58, 59, 64, 106, 109, 110, 111, 112, 113, 114, 115, 116, 117, 118). Moreover, this improvement is pronounced in patients with preoperative sagittal malalignment (20, 33, 35, 52, 56, 112, 115, 118, 119). Advances in surgical techniques, coupled with a growing emphasis on the restoration of sagittal alignment by surgeons during procedures, are likely contributing factors to this trend (31, 102).

The degree of surgical correction in lumbar sagittal alignment is more limited than that achieved in the thoracic region, with studies reporting no statistically significant alteration in lumbar parameters postoperatively (32, 35, 56, 57, 59, 109, 111, 113). However, an increase in LL following surgical intervention has also been observed in a series of clinical studies (58, 64, 110, 116, 120, 121). This may correlate with alterations in TK (33, 50, 52, 102, 116), as improvements in the thoracic region are biomechanically transmitted to the lumbar spine through the thoracolumbar junction and proximal lumbar segments (35, 116, 118, 122).

Surgical intervention significantly improves the sagittal alignment of the cervical spine (32, 33, 34, 35, 58, 59, 64, 109, 111, 113, 114, 115, 117, 120, 122, 123). The degree of cervical improvement is strongly associated with the characteristics of TK (33, 34, 35, 58, 64, 106, 107, 108, 109, 110, 111, 117, 122, 124), and enhanced cervical sagittal alignment has been observed in patients with preoperative sagittal malformation (20, 35, 64, 114). This is consistent with the improvement in thoracic alignment, suggesting a compensatory adjustment mechanism in the cervical spine.

Optimization of sagittal spinal alignment during postoperative follow-up has been observed across the thoracic (108, 118, 124), lumbar (108, 110, 112, 118, 124), and cervical regions (32, 34, 35, 64, 111). This stems from the compensation and coordination between different regions of the spine after surgery; moreover, the self-regulatory mechanisms of the spine and its surrounding muscles and ligaments also come into play. Given that the paraspinal soft tissues in children are undergoing a dynamic developmental process, the supportive and coordinative role of the peri-spinal soft tissues in spinal stability must be considered when formulating surgical strategies.

The SVA maintains stability in patients with AIS, and most studies have demonstrated no significant surgical impact on this parameter (50, 57, 64, 103, 104, 105, 106, 108, 110, 113, 116, 119, 120, 125, 126). Nevertheless, a subset of studies has reported favorable changes in SVA following surgical intervention (35, 52, 58, 109). Studies have documented a transient anterior shift in SVA postoperatively, which subsequently normalized during follow-up (111, 117, 124). This pattern of change is likely attributable to the previously described self-regulatory capacity of the spine in the postoperative adaptive process. Surgical interventions primarily modify regional spinal alignments, yet compensatory coordination among spinal segments and the pelvis sustains global sagittal stability.

The spines of patients with AIS exhibit the capacity to compensate for surgical alterations in spinal curvature (33), thereby ensuring that pelvic morphology remains stable in most cases (10, 32, 33, 35, 48, 50, 57, 64, 109, 111, 113, 119, 120, 122). Studies have observed postoperative pelvic retroversion, manifested as an increase in PT and a decrease in SS (20, 114, 116, 122). This alteration may gradually revert to preoperative levels during the follow-up period, and such changes represent a compensatory mechanism for the tendency toward increased LL following surgery (116, 122, 127).

Despite advances in sagittal reconstruction for AIS, postoperative proximal junctional kyphosis (PJK) remains a critical factor that compromises long-term clinical outcomes. Commonly defined as a proximal junctional angle (PJA) ≥ 10° at final follow-up, PJK reflects a sagittal rebalancing mechanism following surgical alterations in spinal morphology and flexibility (128). Its incidence reaches approximately 20% in AIS cohorts, and severe PJK can manifest as progressive deformity, chronic pain, and neurological deficits, potentially necessitating revision surgery (129, 130). Risk factors include high preoperative TK, overcorrection of LL, use of pedicle screw constructs, and extended fusion lengths (129, 130). Consequently, meticulous preoperative assessment of sagittal parameters, particularly the harmony among TK, LL, and PI, is essential to restore global spinal alignment and effectively mitigate the risk of PJK (128, 129).

### Impact of emerging technologies on sagittal alignment in AIS

Beyond traditional clinical practices, innovative diagnostic and treatment technologies have gained increasing attention in the field of AIS. Evaluating their influence on the spinopelvic sagittal profile is essential to ensure comprehensive three-dimensional correction and ultimately improve long-term prognosis.

In conservative management, current bracing practices increasingly emphasize patient-specific designs tailored to individual spinal morphology. The integration of computer-aided design and manufacturing (CAD/CAM) with patient-specific finite element modeling (FEM) has demonstrated significant biomechanical advantages in recent years. Leveraging patient-specific 3D reconstructions, this technology simulates prefabrication pressure distributions and 3D force vectors to precisely balance coronal correction with sagittal alignment. Research confirms that FEM-optimized braces achieve more effective 3D correction, as for the sagittal plane, the reduction in TK is significantly smaller than that observed with conventional braces (131). Long-term follow-up further indicates that CAD-FEM braces successfully maintain higher LL (132), thereby reducing the risk of flatback deformity. Furthermore, the resulting lighter and thinner design, with reduced surface coverage, may enhance patient compliance and show promising prospects for clinical application (131, 132, 133).

Regarding surgical interventions, studies have indicated that minimally invasive scoliosis surgery (MISS) can achieve outcomes comparable to, or even superior to, those of open procedures in correcting spinal sagittal alignment (134, 135, 136), with the added advantage of reducing surgical complications. While MISS allows enhanced preservation of stability-critical soft tissues, its restricted operative space may compromise the extent of spinal correction (136), warranting further clinical evaluation. Vertebral body tethering (VBT) is an emerging non-fusion surgical technique that harnesses growth potential to achieve deformity correction while preserving segmental mobility. Despite effective coronal correction, its sagittal impact ranges from neutral (137, 138) to a risk of iatrogenic flatback deformity (139), offering no significant advantage over PSF (140). Moreover, it faces challenges of lower corrective power and higher revision rates. Thus, VBT remains reserved for select skeletally immature patients with flexible curves and a strong preference for motion preservation (139). Robotic-assisted spine surgery has also emerged as an area of active investigation, demonstrating advantages such as enhanced accuracy in pedicle screw placement and a shortened learning curve (141, 142, 143). It is hypothesized that these capabilities may translate to favorable sagittal alignment correction, yet this has not been rigorously validated in clinical practice.

Furthermore, the application of artificial intelligence (AI) to measure sagittal parameters has attracted significant attention. Numerous studies demonstrate that deep learning-based models can achieve measurement accuracy comparable to, or even superior to, manual methods while substantially increasing efficiency (144, 145). Although challenges persist in precisely identifying PI and accounting for some anatomical variations (144), this technology has the potential not only to facilitate clinical assessment and treatment planning but also to significantly enhance the efficiency of related clinical research in the future.

## Outlook and conclusion

Despite the synthesized evidence, most current research remains limited by a heavy reliance on single-center studies with follow-up periods typically restricted to 2–5 years. Given the lifelong impact of AIS and the recent emergence of advanced technologies, future multicenter studies with more extended longitudinal tracking are essential to validate the long-term durability of treatment outcomes. Furthermore, because radiographic sagittal alterations do not consistently correlate with clinical QoL, integrating patient-reported outcome measures with imaging metrics is necessary to identify alignment characteristics that truly predict patient satisfaction. Finally, as current evaluation standards are largely derived from adult data, they may not adequately capture the dynamic morphological maturation of the pediatric spine. Establishing age-stratified normative ranges is, therefore, essential to optimize diagnostic and therapeutic precision in patients with AIS.

In summary, sagittal spinal malalignment is a key feature of AIS. The most consistent finding is reduced thoracic kyphosis, often accompanied by compensatory cervical and lumbar changes. Bracing remains effective to control curve progression in the coronal plane, but it carries the risk of inducing flatback deformity, particularly in patients with normal sagittal alignment prior to treatment. Current surgical interventions demonstrate considerable efficacy in improving sagittal alignment, with outcomes positively correlated with the severity of preoperative sagittal imbalance. Despite these regional changes, global sagittal balance tends to remain stable, reflecting the body’s natural effort to preserve overall equilibrium. Although pelvic parameters vary among individuals, pelvic position adapts to maintain spinal balance. Furthermore, emerging technologies have shown promising results in maintaining sagittal spinal balance, and current clinical practice must continue to emphasize the importance of sagittal alignment in the management of AIS.

## ICMJE Statement of Interest

The authors declared no potential conflicts of interest with respect to the research, authorship, and/or publication of this article.

## Funding Statement

The authors received no financial support for the research, authorship, and/or publication of this article.

## Author contribution statement

All authors contributed to the study conception and design. YS and YY were responsible for the initial draft preparation, including the writing and creation of figures. KW and CY provided critical revision of the manuscript for important intellectual content. All authors were actively involved in the process of manuscript development. They all read and approved the final version for submission.
